# Clustering of arc volcanoes caused by temperature perturbations in the back-arc mantle

**DOI:** 10.1038/ncomms15753

**Published:** 2017-06-29

**Authors:** Changyeol Lee, Ikuko Wada

**Affiliations:** 1 Faculty of Earth Systems and Environmental Sciences, Chonnam National University, 33 Yongbong-no Bukgu, Gwangju 61186, Republic of Korea; 2 Department of Earth Sciences, University of Minnesota, 310 Pillsbury Dr. SE, Minneapolis, Minnesota 55455, USA

## Abstract

Clustering of arc volcanoes in subduction zones indicates along-arc variation in the physical condition of the underlying mantle where majority of arc magmas are generated. The sub-arc mantle is brought in from the back-arc largely by slab-driven mantle wedge flow. Dynamic processes in the back-arc, such as small-scale mantle convection, are likely to cause lateral variations in the back-arc mantle temperature. Here we use a simple three-dimensional numerical model to quantify the effects of back-arc temperature perturbations on the mantle wedge flow pattern and sub-arc mantle temperature. Our model calculations show that relatively small temperature perturbations in the back-arc result in vigorous inflow of hotter mantle and subdued inflow of colder mantle beneath the arc due to the temperature dependence of the mantle viscosity. This causes a three-dimensional mantle flow pattern that amplifies the along-arc variations in the sub-arc mantle temperature, providing a simple mechanism for volcano clustering.

## Abstract (web summary)

Volcanoes may cluster along volcanic arcs, but controls on cluster locations are still unclear. Here, using numerical models, the authors show that clustering along arcs is the result of inflow of hotter mantle material, and subdued inflow of colder material creating a 3D mantle flow pattern.

In some parts of subduction margins, such as Northeast (NE) Japan^1^ and Cascadia^2^, arc volcanoes form in clusters (our definition of volcanic clusters excludes those of relatively small monogenetic volcanoes) at variable spacing that ranges from a few tens to hundreds of kilometers. Arc volcanoes are surface expressions of magmatic processes that are induced by plate subduction. In the mantle wedge that overlies the subducting plate, advection of heat by slab-driven mantle wedge flow and addition of aqueous fluids from the subducting slab provide the thermal and chemical conditions required for flux melting^3^,^4^. Therefore, volcanic clustering indicates spatial variation in the physical condition within the mantle wedge. However, the mechanism that causes such spatial variation remains unclear. Understanding the mechanism of volcano clustering is critical to the studies of subduction geodynamics and volcanic hazard.

In NE Japan, the dense seismic networks and abundant earthquakes allow high-resolution imaging of seismic velocity structures, which indicate low-velocity zones (LVZs) in the mantle wedge that extend from the back-arc to the sub-arc region where volcanoes occur in clusters^1^. Because of their strong spatial correlation with volcanic clusters, the LVZs have generally been interpreted as hot regions that feed melts to the overlying clustered volcanoes and have commonly been referred to as hot fingers^1^. Another interpretation of the LVZs is that they represent regions with free fluids and have also been referred to as wet fingers^5^. This interpretation is based on the lack of strong contrast in seismic attenuation between LVZs and the surrounding mantle because temperature is known to have a relatively large effect on seismic attenuation, and high attenuation is expected to occur in hot regions^6^. However, whether the LVZs are hot and/or wet is yet to be verified.

In the mantle wedge, solid-state mantle flow is driven largely by viscous coupling between the subducting slab and the overlying mantle^7^,^8^. This slab-driven mantle wedge flow brings in hot mantle from the back-arc into the sub-arc region. Geophysical observations and numerical modelling indicate that the shallow portion of the mantle wedge is decoupled from the slab, and the trench-ward extent of mantle wedge flow depends on the slab-mantle decoupling depth^9^. Along-arc fluctuation in the slab-mantle decoupling depth is a possible mechanism to cause localized mantle inflow; the overlying mantle is stagnant and cold in regions with deepened decoupling depths whereas the overlying mantle is flowing and hot in regions with shallower decoupling depths^10^. However, this contrasts with global thermal modelling studies that indicate a relatively uniform decoupling depth of 70–80 km (refs 11, 12). Further, detailed thermo-petrologic modelling results for NE Japan with a uniform decoupling depth show a strong correlation between the predicted petrologic structure and the distribution of intraslab earthquakes, supporting the idea that the presence of hydrous minerals or their dehydration is critical for the generation of intermediate-depth intraslab earthquakes^13^. Along-arc fluctuation in the decoupling depth would, however, impact the thermal structure of the subducting slab, requiring an alternative explanation for the intraslab earthquake distribution.

Small-scale mantle convection beneath the arc driven by thermal buoyancy in a low-viscosity mantle wedge is proposed as a possible mechanism for along-arc variation in the sub-arc mantle temperature based on numerical simulations^14^,^15^,^16^. The convection results in alternating regions of mantle upwelling and downwelling, and volcanoes are more likely to form above the regions of hot mantle upwelling, forming volcanic clusters. Another proposed mechanism is the occurrence of isolated small mantle return flow in the mantle wedge^17^. This type of mantle flow has been simulated within a thin low-viscosity layer of 4–8 km thickness imposed at 50–70 km depths immediately above the subducting slab in a numerical subduction model, whereby the along-arc variation in the extent of the return flow is initiated by numerical noise in the thermal field^17^. These mechanisms require relatively low viscosity in the mantle wedge or in a thin mantle layer. Mantle viscosity depends strongly on temperature and water content^18^,^19^. The possible extensive or (vertically) localized occurrence of mantle weakening is attributed to high water content. However, such condition combined with generally high temperature of the convecting mantle is likely to promote widespread melting.

The mantle beneath the arc is brought in from the back-arc by slab-driven mantle wedge flow, and thus the sub-arc mantle temperature depends on the thermal structure of the back-arc. In contrast to the sub-arc region, the relatively large space and vertical temperature variation in the back-arc mantle are likely to promote small-scale buoyancy-driven flow, and this flow has been proposed as the key mechanism that maintains the relatively hot state of the back-arcs that have not undergone significant extension in recent geological history^20^. The upwelling and downwelling of the mantle associated with such buoyancy-driven flow result in lateral temperature perturbations in the back-arc^14^,^16^,^21^. This also results in variations in mantle viscosity, which can affect the dynamics of the subjacent sub-arc mantle. In fact, such effects can easily be simulated in three-dimensional numerical models (see Methods section) using a similar approach to previous studies^14^,^16^,^21^. Further, presence of mantle plumes^22^,^23^ in the back-arc can cause variations in the back-arc mantle temperature, which affect the temperature and viscosity of the mantle that is flowing into the sub-arc region. However, the effects of back-arc temperature perturbations on sub-arc mantle flow and temperature were not investigated previously.

The primary objective of this study is to quantify the effect of back-arc temperature perturbations on the slab-driven mantle wedge flow beneath the arc using a three-dimensional numerical subduction model. In the model, we impose high-temperature anomalies of a specified size and magnitude at a specified depth on the back-arc-side vertical boundary to simulate the effect of back-arc temperature perturbations. The mantle that originates from the high-temperature anomaly is less viscous, resulting in along-arc variations in mantle viscosity and leading to a complex three-dimensional mantle wedge flow pattern that amplifies along-arc variations in sub-arc mantle temperature.

## Results

### Single high-temperature anomaly

**Figure 1:**
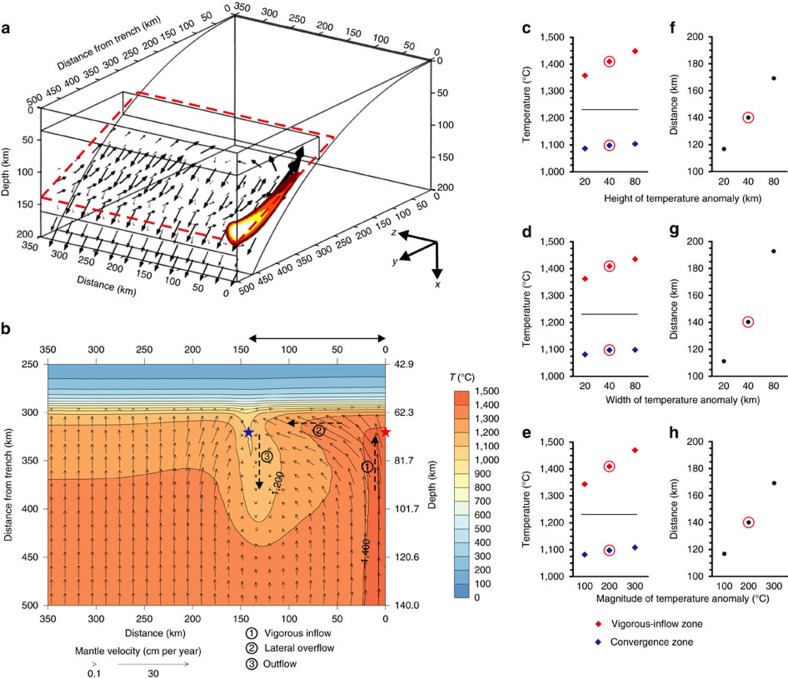
Three-dimensional subduction model with a back-arc high-temperature anomaly. (**a**) Three-dimensional model geometry with one high-temperature anomaly (the reference model). Black arrows indicate the calculated mantle flow field. Colour indicates temperature in the vigorous inflow zone from 1,400 °C (dark red) to 1,500 °C (bright yellow). (**b**) Temperature distribution (colour) and mantle flow field (black vectors) on the dipping plane across the mantle wedge (indicated by the red dashed plane in **a**) calculated from the reference model. The temperature contours are at every 100 °C. The general three-dimensional flow pattern of mantle wedge can be described by combination of vigorous-inflow (1), lateral overflow (2) and outflow in the convergence zone (3). Red and blue stars at a distance of 320 km from the trench indicate the probe locations for mantle temperature in the vigorous-inflow and convergence zones, respectively. (**c**–**e**) Variations in the sub-arc mantle temperature measured at the tip of the vigorous-inflow (red star in **b**) and convergence zones (blue star in **b**) with varying height, width and magnitude, respectively, of the high-temperature anomaly on the back-arc-side vertical boundary. Red circles indicate values from the reference model. Black line indicates the mantle temperature from the model without the high-temperature anomaly. (**f**–**h**) Distance between the vigorous-inflow (red star in **b**) and convergence zones (blue star in **b**) with varying height, width, and magnitude, respectively, of the high-temperature anomaly on the back-arc-side vertical boundary. Red circles indicate values from the reference model.

Simulating the small-scale mantle convection (Supplementary Fig. 1) in the back-arc in the model and quantifying their effects on sub-arc mantle flow and temperature would provide consistency within the model (see Methods). However, simulation of small-scale mantle convection depends on a number of factors, such as the assumed mantle viscosity, vertical temperature and density gradients (through the use of Rayleigh number), and back-arc geometry, and the resulting back-arc temperature perturbations are inherently highly variable in simulations, making it difficult to provide systematic analyses of the effects of back-arc temperature perturbations on sub-arc mantle flow and temperature. For the purpose of this study, therefore, we choose to impose a high-temperature anomaly on the back-arc-side vertical boundary (Fig. 1). The anomaly is represented by an elliptic region defined by its height and width and a prescribed magnitude of increase in temperature towards its center (Supplementary Fig. 2), following the approach developed by Lee and Lim^22^ (See Methods). We use a model with one high-temperature anomaly as our reference model (Fig. 1a); its height and width are 40 km centered at a 120-km depth, and its peak temperature is 200 °C higher than the background mantle potential temperature. In the model, we use a temperature-dependent mantle wedge rheology. The model is symmetric at the right-hand-side side boundary at *z*=0 km, and thus only the left half is shown.

Here, we display the mantle flow and temperature distributions on a dipping plane across the core part of the mantle wedge to illustrate the effects of the high-temperature anomaly in the back-arc (Fig. 1b). Mantle inflow extending from the high-temperature anomaly is more vigorous than the ambient inflow due to its low viscosity. Once the vigorous inflow reaches the wedge corner, some of the mantle is entrained down-dip by the down-going slab as in typical mantle wedge return flow predicted by two-dimensional subduction models, but the rest overflows laterally along the margin, inducing three-dimensional mantle flow. The lateral mantle overflow discourages the ambient inflow, and even in some cases, it leads to shallow outflow of the mantle towards the back-arc (Fig. 1b). This lateral overflow plays a critical role in controlling the mass and heat transfer of sub-arc mantle. Hereafter, we refer to the zone of vigorous inflow that extends from the high-temperature anomaly as the vigorous-inflow zone, and the zone where the lateral overflow and the ambient inflow collide as the convergence zone.

Temperature in the vigorous-inflow zone is elevated relative to that in the ambient sub-arc mantle because the flow originates from the imposed high-temperature anomaly and also due to enhanced vigor of mantle inflow. By contrast, temperature in the convergence zone is reduced as the inflow of hot mantle from the back-arc is subdued by the effect of lateral overflow from the vigorous-inflow zone. This development of the cool convergence zone amplifies the along-arc variations in the sub-arc mantle temperature (Fig. 1c–e). Because of the amplification, a back-arc temperature anomaly that is modest in size and/or magnitude results in relatively large along-arc variations in the sub-arc mantle temperature; for example, a temperature anomaly of 100 °C can result in along-arc temperature variations by ∼250 °C (Fig. 1e). The along-arc variations in the sub-arc mantle temperature increase with the size and magnitude of the high-temperature anomaly (Fig. 1c–e).

**Figure 2:**
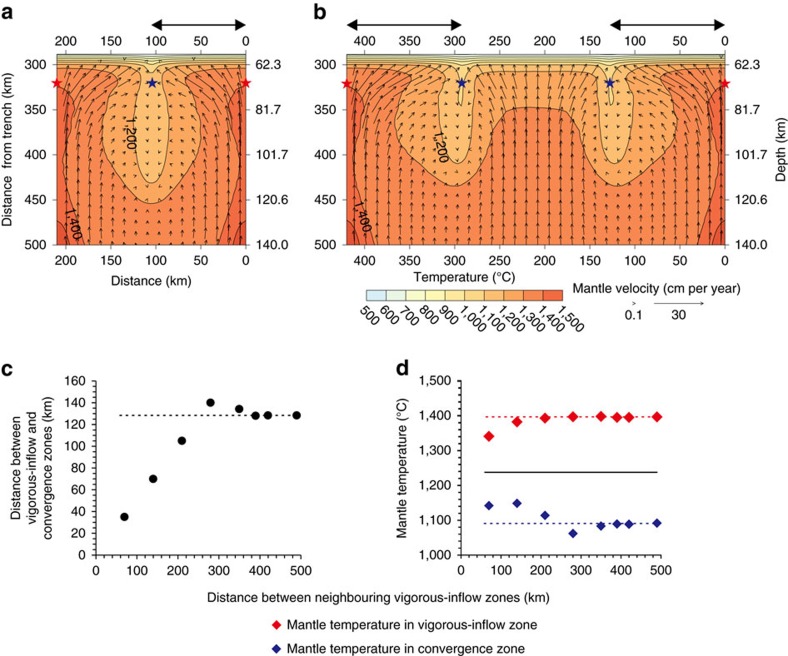
Temperature and mantle flow fields calculated from models with two back-arc high-temperature anomalies. (**a** and **b**) Temperature distribution (colour) and mantle flow field (black vectors) on the dipping plane (indicated by the red dashed plane in Fig. 1a) calculated from models with two high-temperature anomalies that are separated by a distance of 210 and 420 km, respectively. The temperature contours are at every 100 °C. Red and blue stars indicate the probe locations for mantle temperature in the vigorous-inflow and convergence zones, respectively, shown in **d**. Double-arrow-headed lines at the top indicate the distance between the vigorous-inflow and convergence zones. (**c**) Change in the distance between the vigorous-inflow zone (red stars in **a** and **b**) and the convergence zone (blue stars in **a** and **b**) with the distance between the two vigorous-inflow zones (i.e., the two red stars). Black dashed line indicates the characteristic distance (128.3 km) from the reference model. (**d**) The sub-arc mantle temperature at the tip of the vigorous-inflow zones (red diamonds) and convergence zones (blue diamonds). Black line indicates the mantle temperature from the model without the high-temperature anomaly. Red and blue dashed lines indicate the mantle temperatures of the vigorous-inflow and convergence zones, respectively, from the reference model.

A single high-temperature anomaly imposed on the back-arc-side vertical boundary allows the development of the convergence zone at a characteristic distance from the vigorous-inflow zone (Fig. 1f–h). For the reference model, this distance is 140 km. The characteristic distance increases with the size and magnitude of the high-temperature anomaly. In the presence of multiple high-temperature anomalies with small spacing, interference among the vigorous-inflow and convergence zones occurs through the interactions of opposing lateral overflows. Based on a series of simulations varying the distance between two high-temperature anomalies in the back-arc mantle (see Methods section), the vigorous-inflow and convergence zones evolve without interference when the distance between two high-temperature anomalies is about three times the characteristic distance (Fig. 2). Smaller spacing leads to smaller along-arc variations in the sub-arc mantle temperature due to mutual interference (Fig. 2d). For example, when the spacing is about the half of the characteristic distance (that is, 70 km), the resultant along-arc temperature variation is about the same as the magnitude of the imposed temperature anomaly (that is, 200 °C), which is smaller than that without interference (∼310 °C).

**Figure 3:**
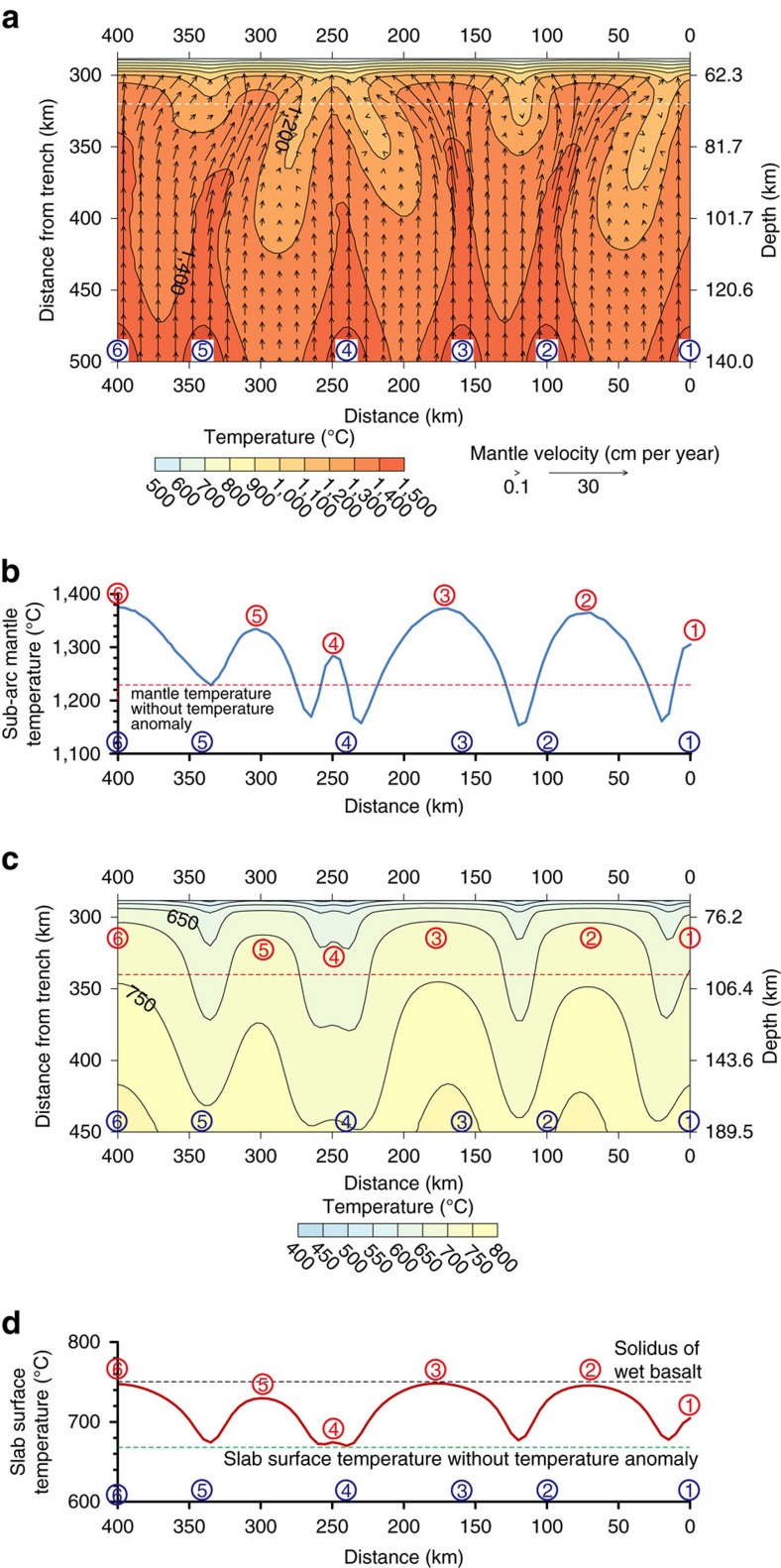
Temperature and mantle flow field calculated from a model with six back-arc high-temperature anomalies. (**a**) Temperature distribution (colour) and mantle flow field (black vectors) on the dipping plane (indicated by the red dashed plane in Fig. 1a) calculated from a model with six high-temperature anomalies at 0, 100, 160, 240, 340 and 400 km distance from the right side-wall (numbered from 1 to 6 enclosed by the blue circles, respectively). The temperature contours are at every 100 °C. (**b**) Temperature (blue line) along the dipping plane at a depth of 70 km (white dashed line in **a**). (**c**) Temperature distribution (colour) on the slab surface. The temperature contours are at every 50 °C. (**d**) Slab-surface temperature (red line) measured at a depth of 100 km (red dashed line in **c**). The black dashed line indicates the solidus of wet basalt^34^,^35^. The green dashed line indicates slab-surface temperature from the model without the back-arc high-temperature anomaly. Numbers enclosed by the blue and red circles in **b**–**d** indicate the locations of the back-arc high temperature anomalies and peak sub-arc mantle temperatures, respectively.

In NE Japan, the distance between the centers of volcanic clusters ranges from ∼50 and ∼120 km with an average of ∼80 km (ref. 1). High-temperature anomalies in the back-arc at this spacing are likely to interfere with one another although the degree of interference depends on the size and magnitude of the anomalies. We impose in our model several high-temperature anomalies with variable spacing ranging from 60 to 100 km to reflect the variability in the volcanic cluster spacing in NE Japan (Fig. 3). The details of the mantle wedge flow pattern depend on a number of other factors, such as the local geometry of the subducting slab and the distribution of fluids and melts, and this model is not intended to reproduce the exact volcano spacing in NE Japan but to provide a better understanding of the effect of possible back-arc temperature anomalies on the overall mantle flow pattern and thermal structure in NE Japan.

The relatively close spacing of the back-arc high-temperature anomalies results in strong interference between the vigorous-inflow and convergence zones (Fig. 3a,b). Further, the non-uniform spacing gives rise to diverse variability in the mantle flow pattern and thermal condition beneath the arc even though all the high-temperature anomalies are of the same size and magnitude. Some of the convergence zones do not extend fully into the sub-arc region or deflected laterally due to the strong interference between neighbouring vigorous-inflow zones, resulting in an along-arc variation in the trench-ward extent and orientation of hot vigorous-inflow zones. The variation in the location of the tip of vigorous-inflow zones is likely to contribute to small along-arc variability of the location of the volcanic clustering within a given subduction zone. The resultant along-arc variations in the sub-arc mantle temperature can give rise to an along-arc temperature variation of ∼100 °C at the sub-arc slab surface (Fig. 3c,d). Our modelling results illustrate the importance of the interference between inflowing mantle of different temperatures and viscosities in controlling the three-dimensional mantle wedge flow pattern and thermal structure of subduction zones.

## Discussion

The three-dimensional mantle wedge flow pattern beneath the arc predicted by our model is characterized by typical corner flow that consists of shallow inflow and down-dip outflow, along-arc lateral overflow, and shallow outflow toward the back-arc. The occurrence of typical corner flow beneath the expected region of volcanism is consistent with the mass transfer from the sub-arc region towards the back-arc inferred from across-arc geochemical variations of arc lavas in NE Japan^24^. The occurrence of lateral overflow and outflow discourages the inflow of hot ambient mantle into the sub-arc region and is likely to contribute to the absence of arc volcanism between regions with the typical return flow.

In our model, the effects of free fluids on mantle wedge flow are not included. Although temperatures in the convergence zones are likely high enough to cause flux melting in the mantle wedge, higher temperatures in the vigorous-inflow zones promote higher degree of partial melting for a given water content^4^. The higher degree of partial melting leads to lower viscosity, which allows more vigorous mantle inflow, and the mantle temperature in the hot inflow zone becomes even higher. Increased vigor of the inflow subdues ambient inflow and results in a cooler condition in the convergence zone. This feedback between temperature, melting, viscosity, and mantle flow is likely to generate temperature contrast between the vigorous-inflow and convergence zones that is larger than what is predicted by our numerical model. This conceptual model provides a simple mechanism for generating relatively large along-arc variations in temperature and melt distributions beneath the arc, satisfying both the hot and wet conditions of the LVZs that are inferred from geophysical observations in NE Japan^1^,^5^.

Previous numerical modelling studies invoked small-scale convection and cold-plumes in the sub-arc mantle driven by thermal and/or compositional buoyancy to explain along-arc variations in the physical conditions of the sub-arc mantle^14^,^16^,^21^,^25^,^26^,^27^. Our modelling results indicate that slab-driven mantle flow alone can cause such variations without invoking buoyancy-driven mantle flow beneath the arc when temperature anomalies are present in the back-arc. This is not to say that there is no small-scale convection or a cold plume, but even in their presence, slab-driven corner flow can dominate the mantle flow field beneath the arc as reported by numerical^28^ and laboratory analog^23^ modelling studies.

Sub-arc mantle temperature strongly influences the thermo-petrologic structure of the upper portion of the subducting slab^29^. At a given depth, the slab beneath the vigorous-inflow zones is hotter than that beneath the convergence zones, experiencing greater degree of dehydration and releasing more fluids into the overlying vigorous-inflow zones. This may lead to lower mantle viscosity and greater degree of flux melting in the mantle wedge, both of which are likely to enhance the vigor of inflow. Further, higher degree of flux melting in the vigorous-inflow zones results in higher permeability than that in the convergence zones^30^, promoting the preferential development of the upward migration pathways. Lower viscosity due to higher temperature and higher water content also facilitates faster fluid migration and melt segregation^31^. These conditions may allow the initial localization of fluids within the vigorous-inflow zone followed by coalescence of fluid transport networks^30^,^32^ that further focuses fluids beneath the clustered volcanoes.

Petrological and geochemical studies of arc lavas in NE Japan indicate little evidence of slab melting^33^. Our model predicts that the slab surface beneath the vigorous-inflow zone (Fig. 3d) is generally cooler than the solidus of wet basalt^34^,^35^. Back-arc temperature anomalies that are greater in size and/or magnitude would result in higher slab-surface temperatures and slab melting. Thus, the size and magnitude of the temperature anomalies are unlikely to be significantly greater than those prescribed in our model.

At the trench-ward end of the LVZs beneath the arc, the S-wave velocity in the mantle becomes relatively uniform along the arc^36^. Slab-driven mantle wedge flow in the presence of back-arc temperature perturbations, small-scale convection or cold plumes beneath the arc cannot explain the uniform sub-arc S-wave velocity, and additional mechanism may be at work, possibly reducing S-wave velocities in the convergence zones and obscuring the along-arc temperature variation, such as the presence of hydrous phases in the cold convergence zones or the effect of seismic anisotropy.

Shear-wave splitting measurements indicate that the sub-arc and forearc mantle in several subduction zones, including NE Japan, exhibit trench-parallel seismically fast polarization directions^37^,^38^. The lattice preferred orientation (LPO) of the olivine fabrics that are dominant in the upper mantle (that is, A- and E-types) develops parallel to the direction of maximum shear^39^. Assuming that the shear direction parallels the mantle flow direction, the trench-parallel mantle flow from the tip of the vigorous-inflow zones towards the convergence zones causes trench-parallel fast polarization direction. The shape preferred orientation (SPO) of partial melts also parallels the shear direction but results in fast polarization direction normal to the shear direction after melt segregation^40^. In vigorous-inflow zones where mantle flows normal to the trench, partial melting and melt segregation can therefore result in trench-parallel fast polarization direction. The combination of the LPO of the laterally flowing mantle and the SPO of partial melts in the vigorous-inflow zones may therefore explain the observed trench-parallel fast polarization direction.

The temperature distribution in the back-arc upper mantle is likely non-uniform as inferred from seismic velocity structures. Although the spatial variations in the back-arc mantle temperature at the length scale of our interest is difficult to constrain at present, our modelling results indicate relatively small high-temperature anomalies of 100–200 °C and a few tens of kilometers in diameter in the back-arc mantle can lead to three-dimensional mantle wedge flow that amplifies the along-arc variations of the sub-arc mantle and slab temperature. In the presence of nonlinear feedback between temperature, viscosity, melting, and mantle flow, the size and magnitude of anomalies in the back-arc that are required to cause such temperature variations are likely even smaller, making it easier for volcano clustering to occur.

## Methods

### Three-dimensional kinematic-dynamic subduction model

Our subduction model^22^ is developed using the commercial finite element package COMSOL Multiphysics. The governing equations consist of equations of conservation of mass, momentum, and energy,


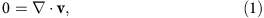



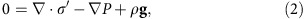



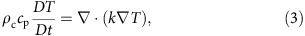


respectively, where **v** is velocity (m s^−1^), *P* is pressure (Pa), *σ′* is deviatoric stress tensor (Pa), *ρ* is density defined as *ρ*=*ρ*_c_ (1−*αT*) (kg m^−3^), *ρ*_c_ is reference density (kg m^−3^), *α* is thermal expansivity (K^−1^), *T* is temperature (K), **g** is gravitational acceleration vector (m s^−2^), *c*_p_ is specific heat at constant pressure (J kg^−1^ K^−1^), *t* is time (s), and *k* is thermal conductivity (W m^−1^ K^−1^). Radiogenic heat production is neglected because its contribution to the thermomechanical behaviour in the subduction zones is relatively small^41^. Viscous dissipation is also neglected; it has been reported that viscous dissipation systematically increases the slab-surface temperature up to ∼50 °C compared with the incompressible model experiments^42^. Equations (1)–(3), 2, 3 are non-dimensionalized using reference values indicated in Supplementary Table 1 for density, temperature contrast, specific heat, thermal conductivity, thermal expansivity, depth of the fluid layer, and viscosity at a depth of 200 km, following the approach of King *et al*.^43^

To illustrate the role of small-scale convection in the back-arc in generating the back-arc temperature perturbations, we first incorporate the effect of buoyancy in a model using the governing equations described above and the Rayleigh number of 2.33 × 10^6^ derived from the model parameter reference values in Supplementary Table 1. The model is 800-km long, 350-km wide and 200-km deep (Supplementary Fig. 1a) and consists of two sub-domains: forearc-arc and back-arc domains. The geometry of the subducting slab is based on the Wadati-Benioff zone estimated by Syracuse and Abers^44^ for NE Japan, but its exact geometry is not critical for this study. The overlying crust is 35-km thick and non-deforming. Subduction velocity of 8.3 cm per year is applied to the slab surface normal to the trench of the model. In NE Japan subduction zone, the Japan trench is largely straight except near its northern and southern ends, and the convergence direction is nearly perpendicular to the trench, a situation similar to our model setup. Previous studies suggested that the corner of the mantle wedge is decoupled from the subducting slab and is stagnant^31^,^44^. To approximate the decoupling effect, we simply impose non-deforming corner to a depth of 70 km (ref. 45).

The trench-side vertical boundary and the bottom boundary of the model are stress-free, and the back-arc-side vertical boundary and side boundaries are insulated and a free-slip. The subducting Pacific plate at the Japan trench is ∼130 Myr old^46^. The geotherm applied to the trench-side vertical boundary and the initial temperature distribution of the entire model domain (including the geotherm applied to the back-arc-side vertical boundary) are calculated by using the half-space cooling model^47^ for a 130-Myr-old and 50-Myr-old plates, respectively, with the mantle potential temperature of 1,350 °C and the mantle adiabat of 0.35 °C km^−1^. The temperature at the top surface of the model is fixed at 0 °C. The basal temperature of the overlying crust in the back-arc domain is fixed at 1,000 °C to account for the relatively thin lithosphere and high surface heat flow (∼88 mW m^−2^) in the back-arc^20^,^48^. We impose a mantle temperature of 1,420 °C at the bottom of the back-arc domain from 550 to 800 km.

For the mantle wedge and the back-arc mantle, we use a diffusion creep rheology,


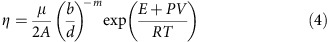


with rheological parameters reported for diffusion creep of dry olivine^18^ (Supplementary Table 2). To approximate the effect of fluid addition from the dehydrating slab into the mantle wedge, the mantle viscosity calculated based on equation (4) is reduced by 1/20 at the corner of the mantle wedge, and the viscosity reduction diminishes linearly with increasing distance away from the corner of the mantle wedge towards the back-arc over ∼120 km distance^21^,^49^. Based on the comparison with the model without the viscosity reduction, we found that the viscosity reduction has a modest effect on the general pattern of mantle wedge flow, and the vigorous inflow, lateral overflow, and outflow to be discussed in the following sections are robust features that result from the presence of back-arc high-temperature anomalies.

The model domain consisted of 969,583 elements including tetrahedral, pyramid, prism, hexahedron, triangular and quadrilateral elements; most of the element are tetrahedrons of which sizes range from 5 to 10 km. Mesh refinements are applied around the boundaries between the mantle wedge and its surrounding sub-domains. The maximum size of the elements in the model is 10.00 km. The mesh around the boundaries between different components of the model is refined, and the smallest size of the element is 2.8 km at the tip of the mantle wedge. Time stepping of the generalized-α is applied to solve the time-evolving governing equations. The Stokes and energy equations are coupled by using the segregated time-dependent solver. For a parallel computation, the Multifrontal Massively Parallel sparse direct Solver (MUMPS) is used. These models with the effect of buoyancy were run for 120 Myr to minimize the effects of initial conditions.

In the model, the subducting slab induces corner flow in the overlying mantle wedge. Because the model includes the effect of thermal buoyancy, the temperature contrast between the top and the bottom of the back-arc mantle drives small-scale convection that generates lateral variations in the shallow mantle temperature (Supplementary Fig. 1d). In the region just behind the arc (at 500 km distance from the trench), the shallow part of the back-arc mantle is dominated by the trench-ward inflow of the back-arc mantle with weakened small-scale convection, and the deeper part is dominated by the down-dip outflow portion of the slab-induced corner flow (Supplementary Fig. 1c). The shallow convection-dominated part of the back-arc mantle is drawn into the forearc-arc region by the corner flow and causes three-dimensional flow beneath the forearc-arc region (Supplementary Fig. 1b). This occurs because the mantle that flows in from the upwelling region in the shallow back-arc mantle is hotter and less viscous than the neighbouring down-welling regions, experiencing more vigorous flow towards the arc. Once the mantle reaches the mantle wedge corner, the vigorous flow spreads laterally to interfere with the neighbouring inflowing mantle, leading to cooler regions of sub-subdued mantle inflow beneath the arc.

This study focuses on the effects of back-arc mantle temperature perturbations on the slab-driven mantle wedge flow pattern. To perform systematic analyses of the effects, we choose to prescribe the back-arc temperature perturbation by imposing a prescribed size and magnitude of high-temperature anomalies at a prescribed depth on the back-arc-side vertical boundary. Since there is no need to simulate small-scale mantle convection in the back-arc, the back-arc domain is removed from the model (Supplementary Fig. 2a), and the back-arc-side vertical boundary is placed at 500 km distance from the trench. Further, in models with a weak mantle wedge that allows small-scale convection, mass and heat transfer in the sub-arc mantle become dominated by the convection, and it is difficult to quantify the effect of back-arc mantle temperature perturbations on the 3D slab-driven mantle wedge flow pattern beneath the arc. Thus, to focus on the slab-driven mantle wedge flow, we exclude the buoyancy term 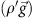 in equation 2. The width of the models is varied depending on the number of high-temperature anomalies imposed on the back-arc-side vertical boundary (discussed below). The geotherm on the back-arc-side vertical boundary is calculated by using the half-space cooling model for a 50-Myr ago plate and is applied from the surface down to a 160-km depth, where the mantle inflow-outflow transition occurs. At depths >160 km, no temperature boundary condition is prescribed. The boundary conditions for the rest of the boundaries of the model are the same as those of the forearc-arc domain in the model with the effect of buoyancy.

On the back-arc-side vertical boundary, we implemented single, two or six high-temperature anomalies defined by the modified distribution function (Supplementary Fig. 2b,c)


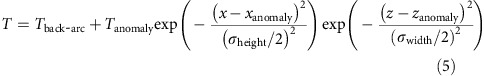


where *T*_back-arc_ is the temperature boundary condition on the back-arc-side vertical boundary (°C); *T*_anomaly_ is the magnitude of the high-temperature anomaly (°C); *x*_anomaly_ and *z*_anomaly_ are the depth from the top surface (km) and distance from the right side-wall boundary (km) of the high-temperature anomaly, respectively; *σ*_height_ and *σ*_width_ correspond to the height and width (km) of the high-temperature anomaly, respectively. At the half of the height and width from the center (red dashed circle in Supplementary Fig. 2c), temperature decreases to 36.79% of the magnitude of the high-temperature anomaly. Slab-driven corner flow causes the hot mantle to flow into the mantle wedge through the high-temperature anomalies on the back-arc-side vertical boundary, causing lower mantle viscosity. As discussed in the main text, the LVZs in the mantle wedge are likely hot and/or wet. Here, we are only explicitly incorporating the temperature effect on mantle viscosity to model hot LVZs. However, wet conditions also reduce mantle viscosity^50^, and the presence of wet LVZs are likely to cause similar effects on mantle wedge flow patterns through mantle viscosity reduction.

The model domain consisted of 255,780 elements including prism, hexahedral, triangular and quadrilateral elements with mesh refinements applied around the boundaries between the mantle wedge and its surrounding sub-domains (Supplementary Fig. 2a). The maximum size of the elements in the mantle wedge is 6.12 km, and that in the rest of the domain is 10 km. The smallest element is 0.4 km at the tip of the mantle wedge. Model calculations with the prescribed back-arc thermal anomalies converged to near steady-state solutions at ∼30 Myr. The models presented in this study were run for 60 Myr to assure a statistical steady state, minimizing the effects of initial conditions.

Temperature anomalies in the back-arc are likely to change in their positions and magnitudes with time. For example, hot upwelling regions caused by small-scale convection may migrate laterally along the arc^14^. While such migration of back-arc temperature anomalies is likely to be critical to periodic repositioning of volcanic clusters, for the purpose of the present study to better understand the first-order effect of back-arc temperature anomalies, we assume stationary back-arc high-temperature anomalies and present the modelling results obtained for a statistical steady-state.

### Single high-temperature anomaly

In the reference model, magnitude (*T*_anomaly_), depth (*x*_anomaly_) and distance (*z*_anomaly_) of the high-temperature anomaly are assumed to be 200 °C, 120 and 0 km, respectively. Both height (*σ*_height_) and width (*σ*_width_) of the high-temperature anomaly are assumed to be 40 km (Supplementary Fig. 2c).

We tested the effect of the height, width, magnitude and depth of the high-temperature anomaly by changing one parameter at a time relative to the reference model (Supplementary Fig. 3). The changes in the sub-arc mantle temperature and the distance between the vigorous-inflow and convergence zones with the height, width, and magnitude of the anomaly are summarized in Fig. 1c–h, and those with the depth of the anomaly are shown in Supplementary Fig. 4. In these figures, the maximum and minimum sub-arc mantle temperatures at a depth of 70 km and a distance of 320 km from the trench occur in the vigorous-inflow (right black star in Fig. 1b) and convergence zones (left black star in Fig. 1b), respectively. As discussed in the main text, an increase in height, width and magnitude of the high-temperature anomaly results in greater influx of the hot mantle into the mantle wedge, leading to higher sub-arc mantle temperatures and a larger distance between the vigorous-inflow and convergence zones. The velocity at which the mantle enters from the back-arc into the mantle wedge varies with depth. When the depth of the high-temperature anomaly coincides with peak mantle inflow velocities at the back-arc-side vertical boundary, the sub-arc mantle temperatures and the distance between the vigorous-inflow and convergence zones become maximized due to the high temperature and great vigor of the inflow. We found that the peak mantle inflow on the back-arc-side vertical boundary occurs at about 120 km depth (Supplementary Fig. 4b). Deviation of the high-temperature anomaly from this depth results in reductions in the sub-arc mantle temperatures and the distance between the vigorous-inflow and convergence zones, but the effect is relatively small.

The resultant complex temperature field in the mantle wedge leads to along-arc variations in the slab-surface temperature. The maximum and minimum slab-surface temperatures under the vigorous-inflow and convergence zones, respectively, at a depth of 100 km and a distance of 340 km from the trench are shown in Supplementary Fig. 5. Changes in the slab-surface temperature with the aforementioned parameters follow the same trends observed for sub-arc mantle temperatures.

### Two high-temperature anomalies

Here, we implemented two back-arc high-temperature anomalies centered at the right and left side-wall boundaries and evaluated their interference (Fig. 2a,b). We changed the spacing between the two high-temperature anomalies by changing the width of the model domain. Those with spacing of 210 and 420 km are shown in Fig. 2a,b, respectively, and the results of those with other spacing are summarized in Fig. 2c,d. In these models with two high-temperature anomalies, the depths of both temperature anomalies are set to 140 km. The other parameters that define the high-temperature anomalies except for the depth are the same as those used for the reference model.

For spacing of < ∼250 km, the lateral overflows from the two vigorous-inflow zones merge together to form a single convergence zone between the two vigorous-inflow zones, and therefore, the distance between the vigorous-inflow and convergence zones is half of the spacing (Fig. 2c). The interference between the two lateral overflows results in the development of less prominent vigorous-inflow and convergence zones, and the temperature contrast between the two zones is smaller than in the reference model (Fig. 2d). For spacing of >∼250 km, the lateral overflows from the two vigorous-inflow zones do not interfere with each other, and they each form a convergence zone at a characteristic distance (128.3 km) from the respective vigorous-inflow zone (Fig. 2c). When the spacing is ∼280 km, the largest temperature contrast (338.4 °C) occurs between the vigorous-inflow and convergence zones, and as we increase the spacing to 390 km, the temperature contrasts converges to 310.2 °C (Fig. 2d), the same temperature contrast observed in the reference model. These results indicate that the two vigorous-inflow zones develop independently when the spacing is larger than 390 km, around three times the characteristic distance of the vigorous-inflow and convergence zones.

As shown in the model using a single high-temperature anomaly, slab-surface temperature reflects the along-arc variations in the sub-arc mantle temperature (Supplementary Fig. 6). Because of the subdued development of the vigorous-inflow and convergence zones at spacing of <280 km, the temperature contrast between the slab surfaces under the vigorous-inflow and merged convergence zones is smaller than that in the reference model. The temperature contrast between the slab surfaces under the vigorous-inflow and merged convergence zones is maximized when the distance between the two high-temperature anomalies is 280 km (Supplementary Fig. 6c). When the distance between the two high-temperature anomalies is the three times the characteristic distance between the vigorous-inflow and convergence zones, the vigorous-inflow and convergence zones independently develop (Supplementary Fig. 6b).

### Six high-temperature anomalies

We impose six high-temperature anomalies on the back-arc-side vertical boundary at 0, 100, 160, 240, 340 and 400 km from the right side-wall boundary of the model, with the spacing of 100, 60, 80, 100 and 60 km (Fig. 3a). The depth of temperature anomalies is set to 140 km. The other parameters that define the high-temperature anomalies are the same as those used for the reference model.

The strong interference between the vigorous-inflow and convergence zones occurs in this model (Fig. 3a). For example, the vigorous-inflow zones that extend from Anomalies 2 and 3 are deflected away from each other (Fig. 3b). In general, greater degree of deflection occurs when spacing between high-temperature anomalies is narrower. The vigorous-inflow zone that extends from Anomaly 5 is deflected significantly to the right due to the vigorous-inflow zone that develops from Anomaly 6 along the side boundary at *z*=400 km with no-flow boundary condition. The interaction among vigorous-inflow zones and development of convergence zones results in complex mantle flow and temperature distributions. The vigorous-inflow zones that extend from Anomalies 2, 3 and 6 result in high sub-arc mantle temperatures, which in turn cause high slab-surface temperatures, compared with those beneath the other vigorous-inflow zones.

### Data availability

The model input required to reproduce the results are available within the article. The modelling results referenced in this study are available on request from C.L.

## Additional information

**How to cite this article:** Lee, C. & Wada, I. Clustering of arc volcanoes caused by temperature perturbations in the back-arc mantle. *Nat. Commun.* **8,** 15753 doi: 10.1038/ncomms15753 (2017).

**Publisher’s note:** Springer Nature remains neutral with regard to jurisdictional claims in published maps and institutional affiliations.

## Supplementary Material

Supplementary Information Supplementary Figures and Supplementary Tables
