# Supplementary material for: Clustering of arc volcanoes caused by temperature perturbations in the back-arc mantle
Source: Nat Commun. 2017 Jun 29;8:15753. doi: 10.1038/ncomms15753 (PMC5493751; doi:10.1038/ncomms15753)
Supplement: Supplementary Information — Supplementary Figures and Supplementary Tables [file ncomms15753-s1.pdf]

Supplementary Information

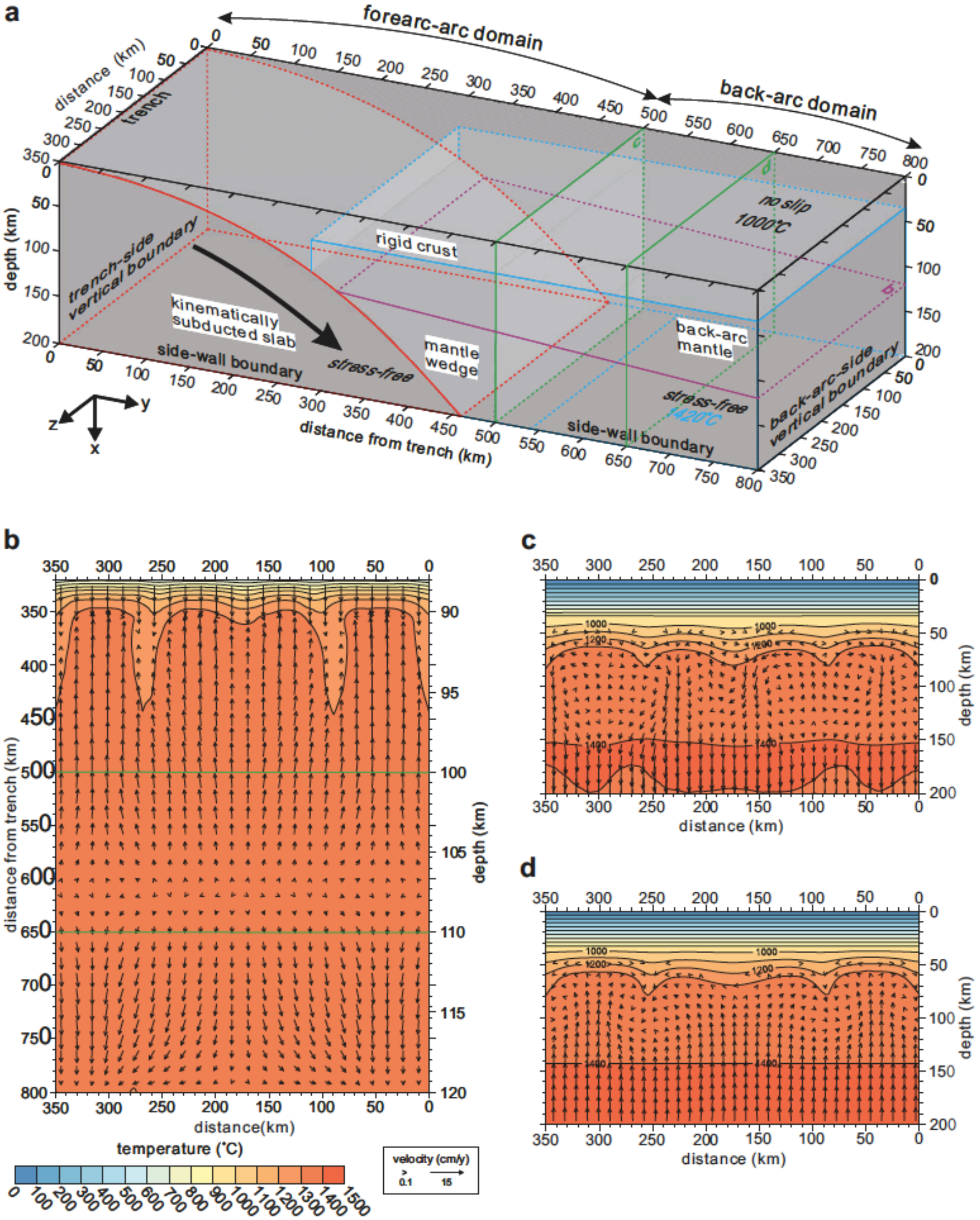

Supplementary Figure 1. | Three-dimensional model construction with the effect of thermal buoyancy. a, Three-dimensional model geometry consisting of the forearc-arc and back-arc domains with model boundaries. The motion of the subducting slab is kinematically prescribed, and the overlying mantle is dynamically controlled by slab-driven mantle wedge flow and buoyancy-driven mantle flow. Thick black arrow indicates the subduction direction. The crust on the subducting slab, mantle wedge and back-arc mantle is non-deforming (rigid). Planes indicated by purple and green lines correspond to the dipping and vertical

cross sections shown in **b**, **c** and **d**, respectively. Mantle flow field (black vectors) and temperature distribution (color) along **b**, a dipping cross section and vertical cross sections at **c**, 500 km and **d**, 650 km distances from the trench. Green lines in **b**, indicates the location of the vertical cross sections at 500 and 650 km distances from the trench. The temperature contours are at every 100°C. The temperature anomalies created by the small-scale convection in the back-arc mantle generate three-dimensional mantle flow pattern beneath the arc.

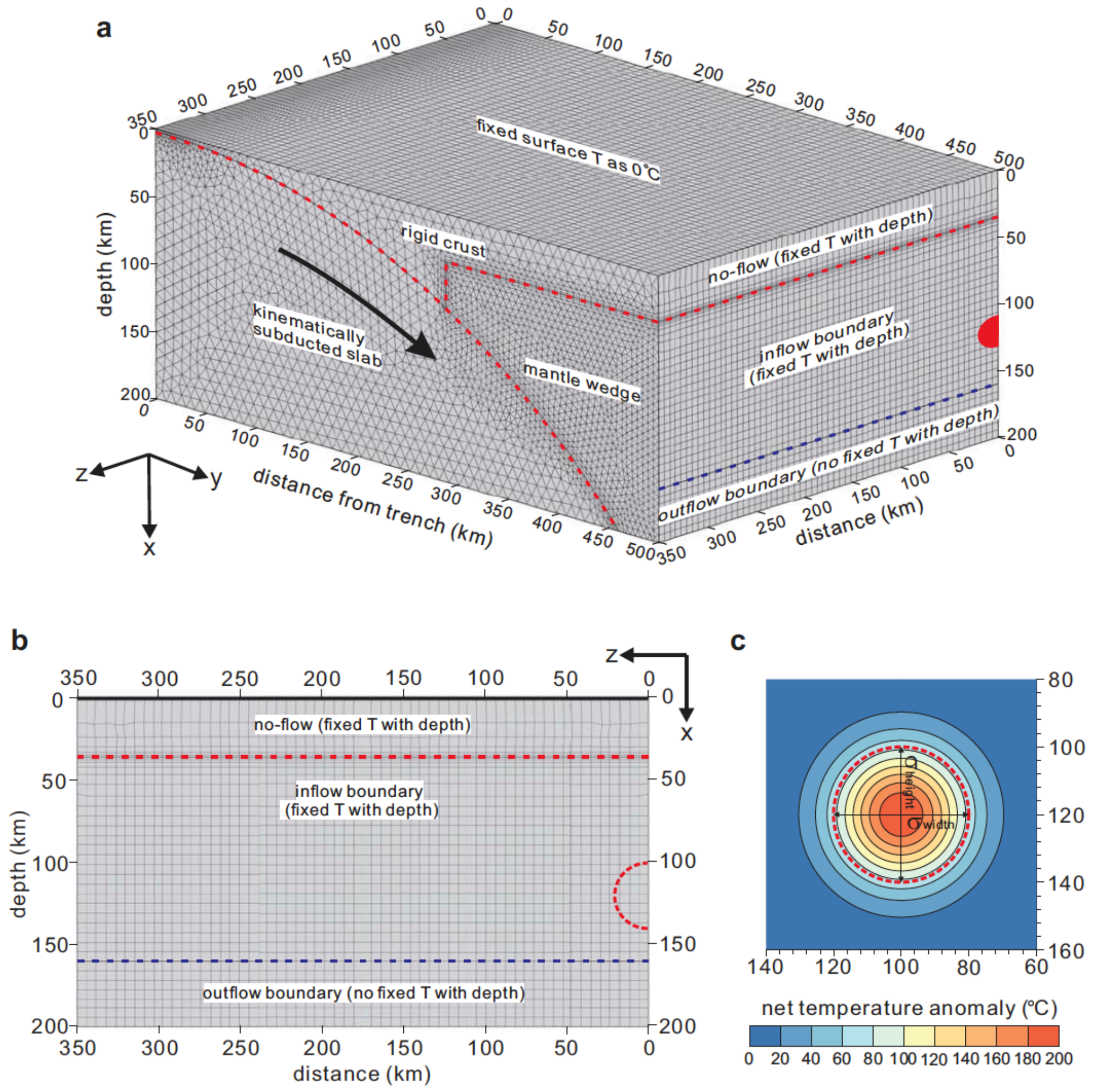

Supplementary Figure 2. | Three-dimensional model construction with an imposed back-arc high-temperature anomaly. a, Three-dimensional model geometry with finite element mesh shown on model boundaries. The motion of the subducting slab is kinematically prescribed, and the overlying mantle is dynamically advected by slab-driven mantle wedge flow. Thick black arrow indicates the subduction direction. Red dashed lines indicate the boundaries between the three sub-domains: the subducting slab, mantle wedge, and rigid overlying crust. Red ellipse on the back-arc-side vertical boundary indicates where the high-temperature anomaly is imposed in the reference model. Blue dashed line indicates the depth to which the back-arc geotherm is applied, corresponding roughly to the depth of the mantle inflow-outflow transition. **b,** Temperature boundary conditions for the back-arc-side vertical boundary for the reference model. **c,** Temperature distribution of the high-temperature anomaly. σ_{height} and σ_{width} correspond to the height and width of the high-temperature anomaly defined by a modified distribution function; both height and width are 40 km in the reference model. Red dashed circle in **b** and **c** corresponds to the location where the temperature is 36.79 % (73.58°C) of the temperature magnitude (T_{anomaly}, 200°C).

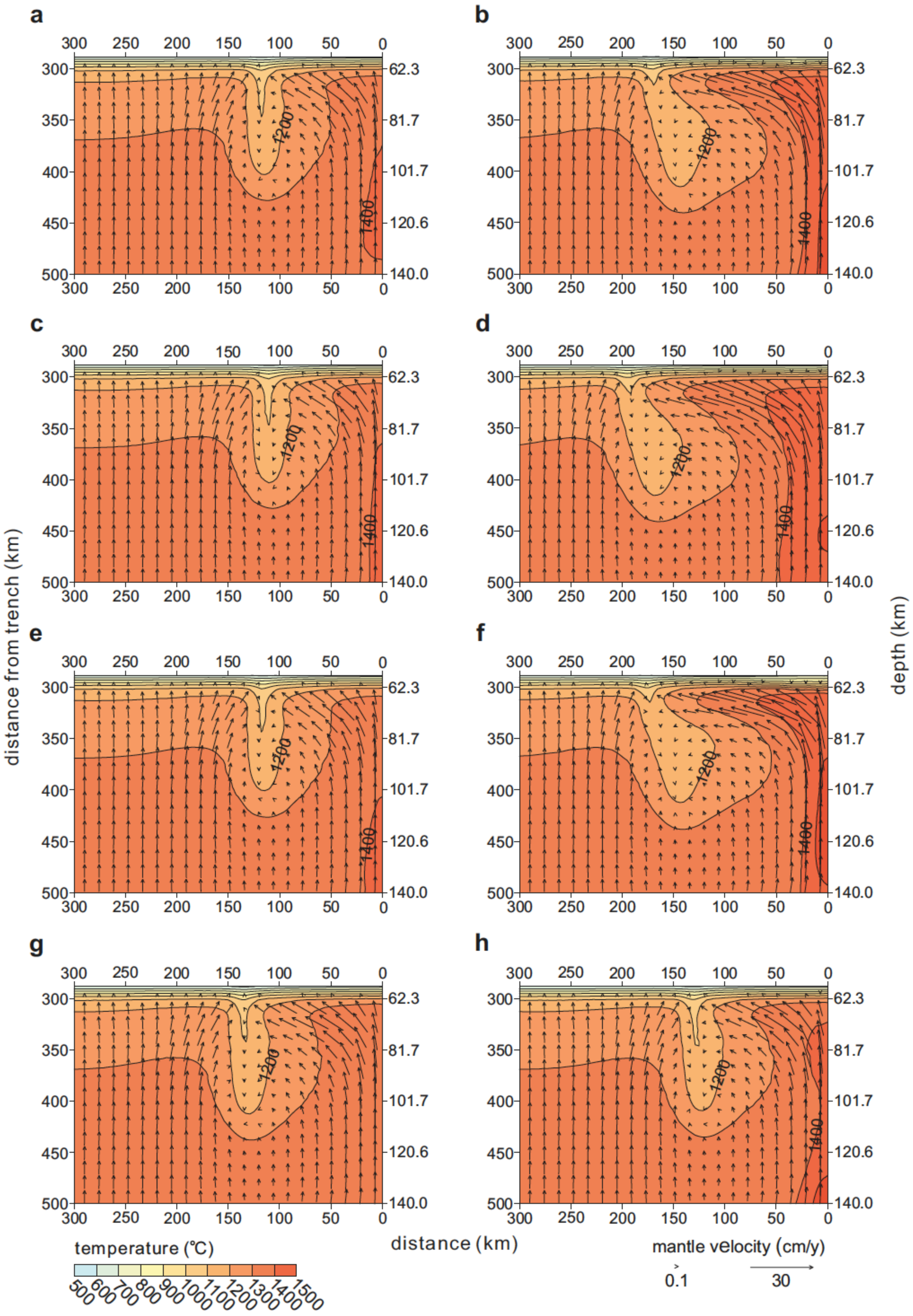

Supplementary Figure 3. | Effects of the height, width, magnitude, and depth of a high-temperature anomaly on mantle flow and temperature distribution. Temperature distribution (color) and mantle flow fields (black vectors) on the dipping plane (indicated by

the red dashed plane in Fig. 1a) calculated by varying the height, width, magnitude, and depth of the high-temperature anomaly. The model geometry is the same as the reference model, but only the region within 300 km of the right side-wall boundary is shown. **a** and **b,** Shorter and taller anomalies (20 and 80 km). **c** and **d,** Narrower and wider anomalies (20 and 80 km). **e** and **f,** Cooler and hotter anomalies (100 and 300°C). **g** and **h,** Shallower and deeper anomalies (100 and 140 km), relative to that in the reference model. The temperature contours are at every 100°C.

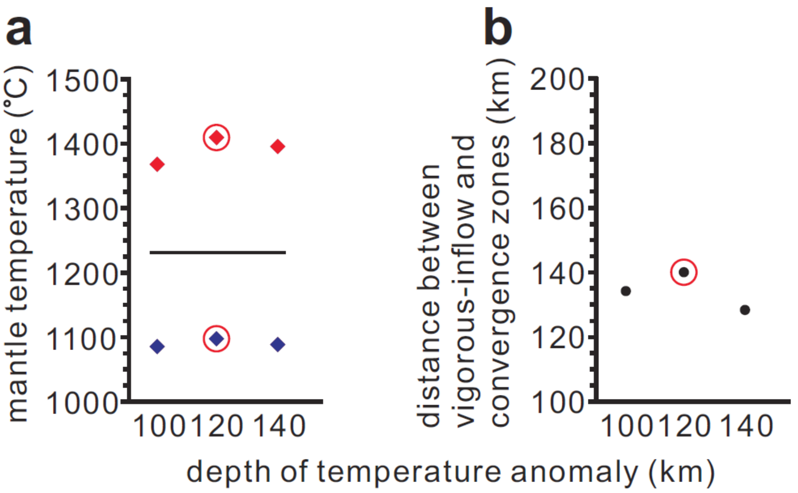

Supplementary Figure 4. | Mantle temperatures of the vigorous-inflow and convergence zones, and the distance between the vigorous-inflow and convergence zones with the depth of the high-temperature anomaly. a, Mantle temperatures at the tip of the vigorous-inflow zone (red diamod) and convergence zone (blue diamond). Red circles indicate mantle temperatures from the reference model. Black line indicates mantle temperatures from the model without the high-temperature anomaly. **b**, Distance between the vigorous-inflow and convergence zones. Red circle indicates the distance from the reference model.

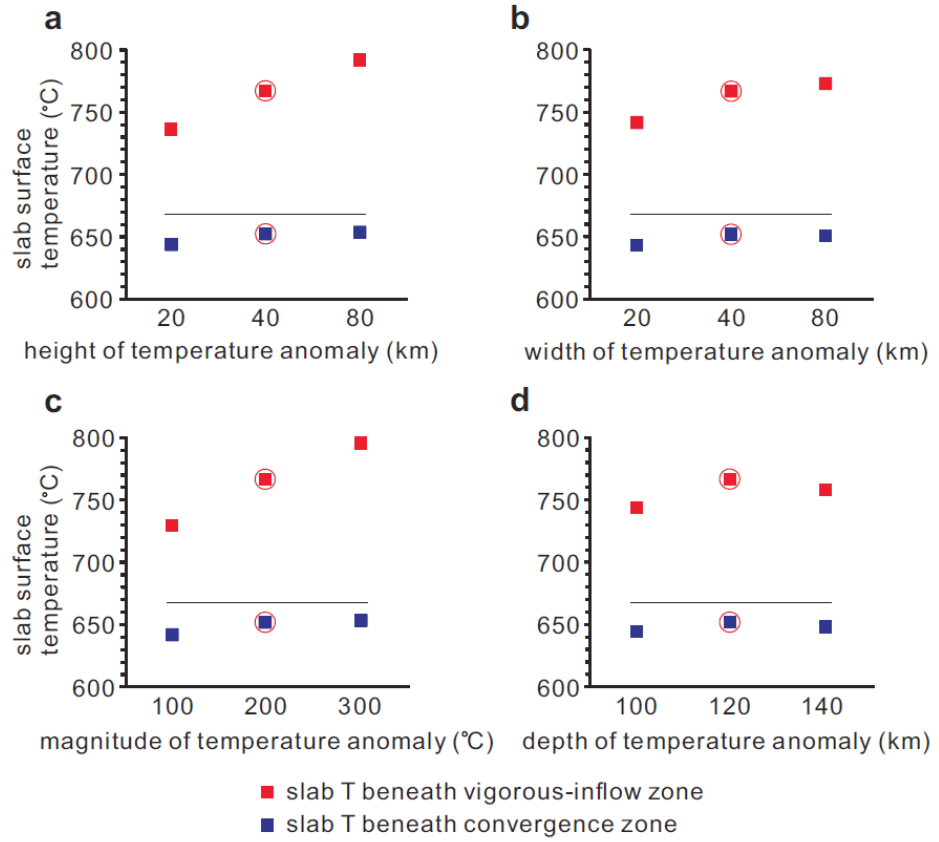

Supplementary Figure 5. | Effects of the height, width, magnitude, and depth of a high-temperature anomaly on slab-surface temperatures. a–d, Slab-surface temperatures beneath the vigorous-inflow zone (red square) and convergence zone (blue square) with varying height, width, magnitude, and depth, respectively, of the high-temperature anomaly. Red circles indicate slab-surface temperatures from the reference model. Black line indicates slab-surface temperature from the model without the high-temperature anomaly.

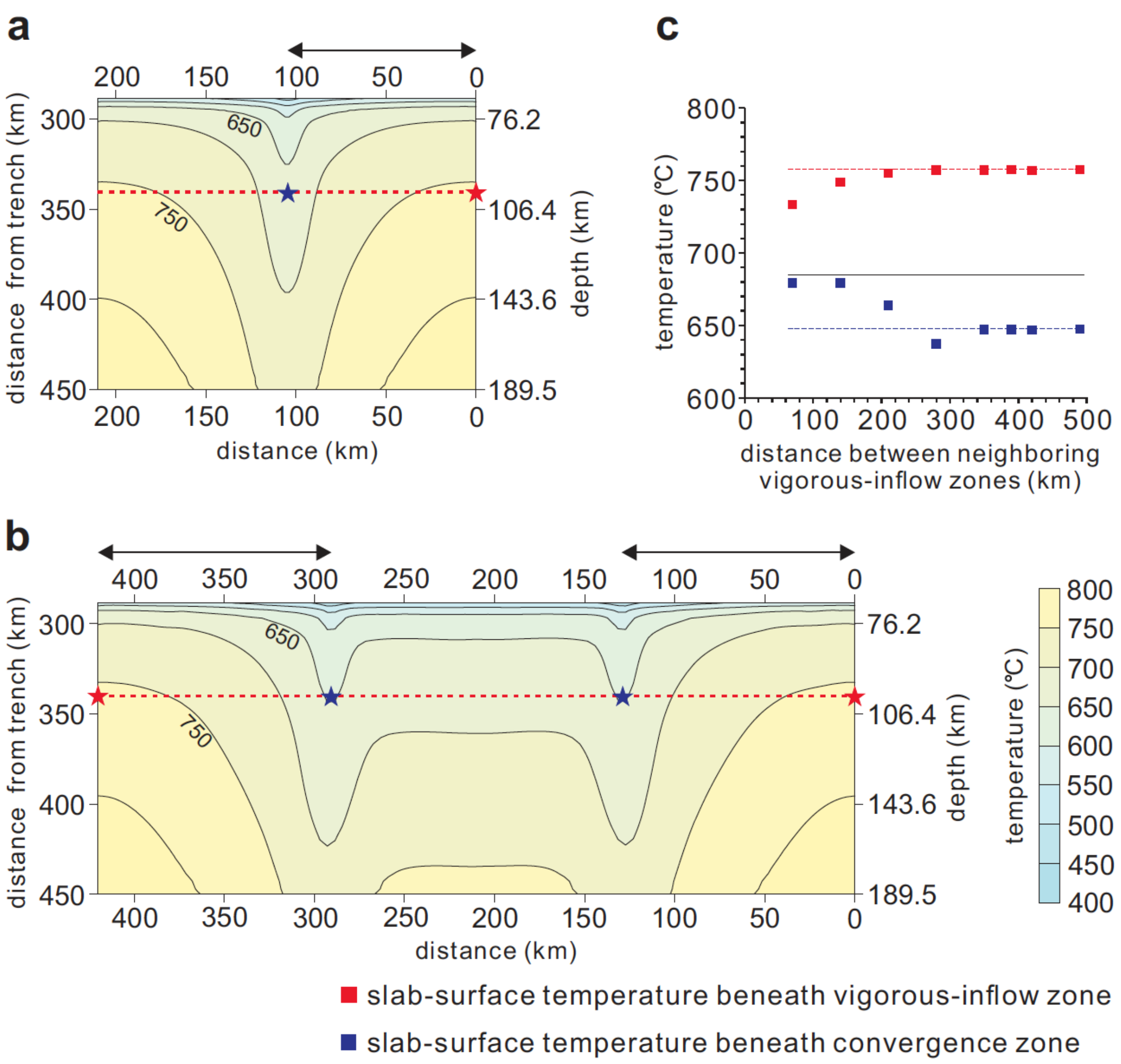

Supplementary Figure 6. | Slab-surface temperature with two high-temperature anomalies. a and **b,** Temperature distribution (color) on the slab surface calculated from models with two high-temperature anomalies that are separated by spacing of 210 km and 420 km, respectively. The temperature contours are at every 50°C. Red dashed line corresponds to a depth of 100 km on the slab surface. **c,** Slab-surface temperatures at a depth of 100 km beneath the vigorous-inflow zones (red square) and convergence zones (blue square) calculated from models with different spacing of the two high-temperature anomalies. Red and blue dashed lines correspond to the slab-surface temperatures beneath the vigorous-inflow and convergence zones, respectively, from the reference model. Black dashed line corresponds to the slab-surface temperature from the model without the high-temperature anomaly.

Supplementary Table 1. Model parameters and their reference values

Surface temperature (K)	273
Density (kg m^{-3})	3300
Specific heat (J kg^{-1} K^{-1})	1200
Thermal conductivity (W m^{-1} K^{-1})	3.0
Thermal expansivity (K^{-1})	2.5×10^{-5}
Depth of the fluid layer in the back-arc (km)	165
Temperature contrast in the back-arc (K)	420

Supplementary Table 2. Rheological parameters and their values

Shear modulus μ (Pa)	8.0×10^{10}
Pre-exponential factor A (s^{-1})	8.7×10^{15}
Burgers vector b (m)	5.0×10^{-10}
Grain size d (m)	1.0×10^{-3}
Grain size exponent m	2
Activation energy E (J mol^{-1})	300×10^{3}
Activation volume V (m^3 mol^{-1})	6.0×10^{-6}
Gas constant R (J mol^{-1} K^{-1})	8.314
